# Multidrug resistance of *Botrytis cinerea* associated with its adaptation to plant secondary metabolites

**DOI:** 10.1128/mbio.02237-23

**Published:** 2024-01-23

**Authors:** Zhaochen Wu, Yue Bi, Junting Zhang, Tuqiang Gao, Xueming Li, Jianjun Hao, Guihua Li, Pengfei Liu, Xili Liu

**Affiliations:** 1 Department of Plant Pathology, China Agricultural University, Beijing, China; 2 Department of Plant Pathology, Tianjin Agricultural University, Tianjin, China; 3 School of Food and Agriculture, University of Maine, Orono, Maine, USA; 4 College of Plant Sciences, Jilin University, Changchun, China; Case Western Reserve University School of Medicine, Cleveland, Ohio, USA

**Keywords:** ATP-binding cassette (ABC), preadaptation, fungicide resistance, MDR development

## Abstract

Fungicides are an effective way to control gray mold of grapes, but the pathogen *Botrytis cinerea* can develop resistance, overcoming the effectiveness of a fungicide that is repeatedly applied. More importantly, the emergence of multidrug resistance (MDR) in the field, where multiple fungicides with different modes of action simultaneously lose their efficacies, is a significant concern. MDR is associated with ATP-binding cassette (ABC) transporters of the pathogen, and certain plant secondary metabolites (PSMs) stimulate the upregulation of ABC transporters, we hypothesized that the pathogen’s preadaptation to PSMs might contribute to MDR development. To test this in *B. cinerea,* ten PSMs, namely, resveratrol, reserpine, chalcone, flavanone, eugenol, farnesol, anethene, camptothecin, salicylic acid, and psoralen, were selected based on their association with ABC transporters involved in fungicide resistance. *B. cinerea* strain B05.10 was continuously transferred for 15 generations on potato dextrose agar amended with a PSM (PDAP), and sensitivities to PSMs and fungicides were examined on the 5th, 10th, and 15th generations. RNA was extracted from *B. cinerea* from the selected generations. After 15 generations of culture transfers, an up-regulation was observed in the expression of ABC transporter-encoding genes *BcatrB, BcatrD,* and *BcatrK* using quantitative polymerase chain reaction (qPCR). This upregulation was found to contribute to MDR of *B. cinerea* against two or more fungicides, among azoxystrobin, boscalid, fludioxonil, difenoconazole, prochloraz, and pyrimethanil. This finding was confirmed through genetic transformation. The decreased sensitivity of *B. cinerea* to fungicides was confirmed as a subsequent MDR phenotype after exposure to camptothecin, flavanone, and resveratrol. Besides, transcriptome analysis also revealed the upregulation of transcription factors related to ABC expression following resveratrol exposure. This suggests that PSMs contributed to inducing preadaptation of *B. cinerea,* leading to subsequent MDR.

**IMPORTANCE:**

The emergence of MDR in plant pathogens is a threat to plant disease management and leads to the use of excessive fungicides. *Botrytis cinerea* is of particular concern because its MDR has widely emerged in the field. Understanding its genesis is the first step for controlling MDR. In this study, the contribution of PSMs to MDR has been examined. Effective management of this pathogen in agroecosystems relies on a better understanding of how it copes with phytochemicals or fungicides.

## INTRODUCTION

*Botrytis cinerea* is a pathogen that causes significant damage to agricultural production, and some of the diseases caused by them are devastating. Fungicides have been extensively used to control the diseases caused by *B. cinerea*. Unfortunately, frequent applications of fungicides have resulted in the resistance of *B. cinerea* to these chemicals. More importantly, multidrug resistance (MDR) has emerged in *B. cinerea* in the field (1, 2). This indicates that pathogens can develop resistance to several fungicides that possess different modes of action (MOA) simultaneously. This occurrence may lead to the failure of disease management through a conventional resistance management strategy, such as alternating or tank-mixing of fungicides with different MOAs. The excessive use of fungicides in field crops can multiply environmental pollution and increase the risk of food contamination due to pesticide residues.

There are various known mechanisms of fungicide resistance, including genetic mutation, overexpression of the target protein, xenobiotic metabolism (3), and activation of efflux pumps, such as ATP-binding cassette (ABC) transporters and major facilitator superfamily (MFS) transporters (4). Efflux-related resistance is the most important mechanism in *B. cinerea*. The ABC transporter is one of the largest transporter families, which uses the energy produced by ATP hydrolysis (5). The two core structures of ABC transporters usually consist of six trans-membrane domains and two nucleotide-binding fold domains. Fourteen ABC transporters (*BcatrA* to *BcatrN*) have been identified (6). Among them, the *BcatrB, BcatrD*, and *BcatrK* genes have been reported to be associated with MDR in *B. cinerea* (7–9).

Plant secondary metabolites (PSMs) can influence both pathogens and plants in their interactions. While they can help in defending plants against pathogen infections, they may also elicit the pathogen to overcome the defense of plants. Most studies have been focused on PSMs involved in phytoalexin production (10), but how pathogens defend themselves against PSMs has been rarely investigated. Some PSMs have been found to affect ABC transporters of pathogens by enhancing the expression of ABC genes (11–16). For example, resveratrol induces the overexpression of the *atrB* gene in *B. cinerea* (11) and of the *PMR5* and *Bcmfs1* genes in *Penicillium digitatum* (12), while psoralen and eugenol induce *Bcmfs1* gene overexpression in *B. cinerea* (13). Camptothecin induces *atrB* overexpression in *Aspergillus nidulans* (14). The alkaloid reserpine can cause upregulation of ABC transporters’ gene expression in *Aspergillus oryzae* (15). Chalcone induces the overexpression of P-glycoprotein (P-gp) and multidrug resistance proteins (16). Meanwhile, some other PSMs inhibit the function of ABC transporters. Flavanone, farnesol, chalcone, and anethene are transporter protein inhibitors (16, 17), and beauvericin counteracts MDR by intercepting the ABC transporters (18). Ellagic acid and schisandrins derivatives, which are natural biannual polyphenols, have therapeutic potential in overcoming MDR in cancer by regulating drug efflux through P-gp and similar transporters (19). Transporters in pathogens are key factors contributing to multidrug efflux, which caused changes in pathogen sensitivity to plant-derived secondary metabolites. Thus, ABC transporters may regulate the adaptability of pathogens to xenobiotic compounds, such as PSMs and fungicides (11).

ABC transporters play a critical role in the adaptability of pathogens to xenobiotic compounds such as PSMs and fungicides (11). These transporters also influence the interaction between pathogens and antibiotics, including 2,4-diacetylphloroglucinol, phenazine-1-carboxylic acid, and phenazine-1-carboxamide, broad-spectrum antibiotics produced by *Pseudomonas* spp. (20) . A plant pathogenic fungi may gradually build up resistance to the antibiotic pyrrolnitrin over time, rendering it less effective against biocontrol agents that target *B. cinerea* (21).

While it is unclear whether the overexpression of ABC transporter genes induced by PSMs can result in MDR in *B. cinerea*, it is possible that the preadaptation of plant pathogens may contribute to MDR observed in the field. Previous studies suggest that such preadaptation may be linked to the preadaptive reactions to PSMs existing in human pathogens (22) or insects (23). In our previous study, it was found that the PSMs such as resveratrol accumulated significantly in grape leaves infected by *B. cinerea* (24). Whether these PSMs participated in the preadaptation of pathogens to fungicides remains unclear. Thus, the objectives of this study were to examine whether PSM preadaptation exists in *B. cinerea* and whether it even contributes to MDR. The results will help us understand the mechanisms of development of MDR in the field.

## RESULTS

### Adaptation of *Botrytis cinerea* to PSM stress

**Fig 1:**
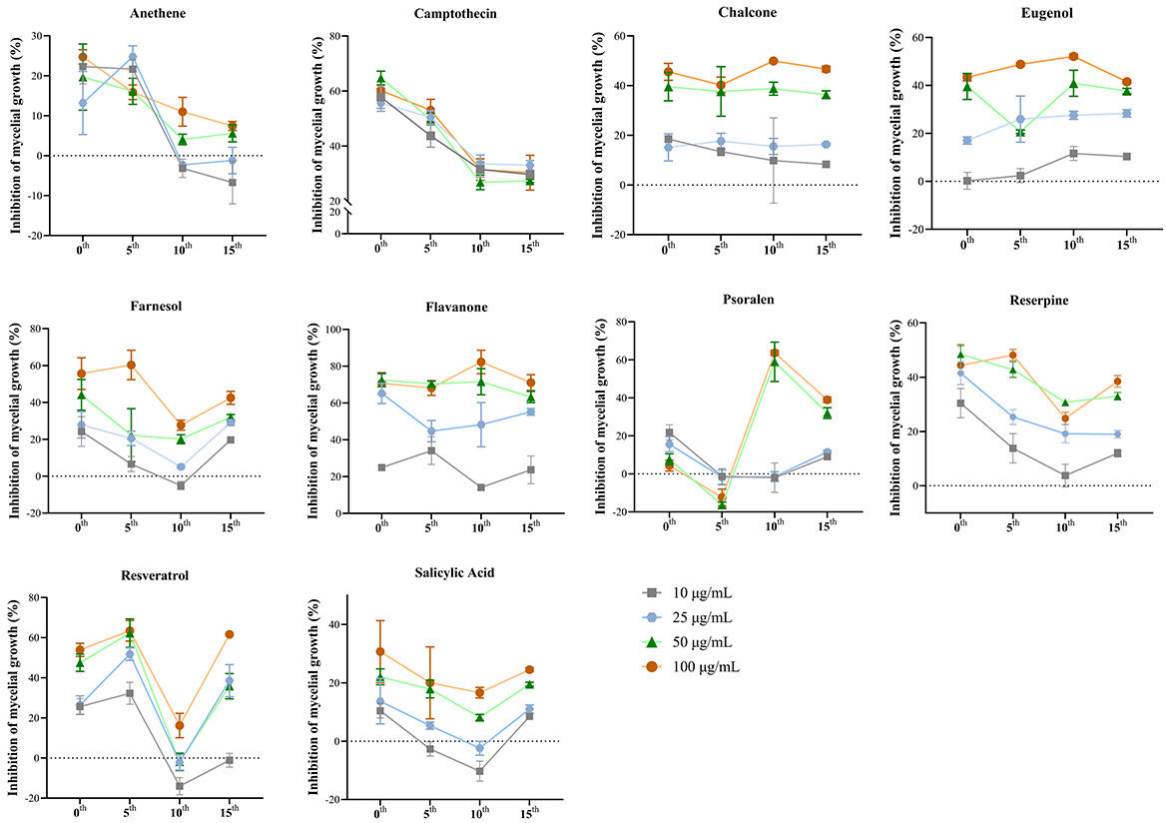
Sensitivity of plant secondary metabolite (PSM)-adapted *Botrytis cinerea* B05.10 to PSMs at the 0 (parental culture), 5th, 10th, and 15th culture transfers or generations, measured by mycelial growth inhibition.

A series of culture transfers of *B. cinerea* was performed on potato dextrose agar amended with a PSM (PDAP) to establish a condition for preadaptation. The sensitivity was determined on the parents, and the 5th, the 10th, and the 15th generations of *B. cinerea* B05.10 were exposed to PSMs, and the decreased inhibition of induced mutants was compared to that of B05.10 (Fig. 1).

There were four distinct sensitivity patterns observed in *B. cinerea* when exposed to PSMs, with a few exceptions. Pattern 1: sensitivity continuously declined during the initial ten transfers, stabilizing or increasing thereafter. This pattern was observed in treatments with anethene, camptothecin, farnesol, reserpine, and salicylic acid. Pattern 2: sensitivity slightly increased at the 5th transfer, then significantly decreased at the 10th transfer, and eventually returned to the 5th transfer level. Only the resveratrol treatment followed this pattern. Pattern 3: sensitivity declined at the 5th transfer, significantly increased at the 10th transfer, and decreased again at the 15th transfer. This was observed with psoralen treatment, displaying Pattern 1 at lower concentrations (≤25 µg/mL) and Pattern 3 at higher concentrations (≥50 µg/mL). Pattern 4: sensitivity remained relatively stable across all 15 transfers for chalcone, eugenol, and flavanone treatments.

### Sensitivities of the PSM-adapted mutant of *Botrytis cinerea* to multiple fungicides with different modes of action

**Fig 2:**
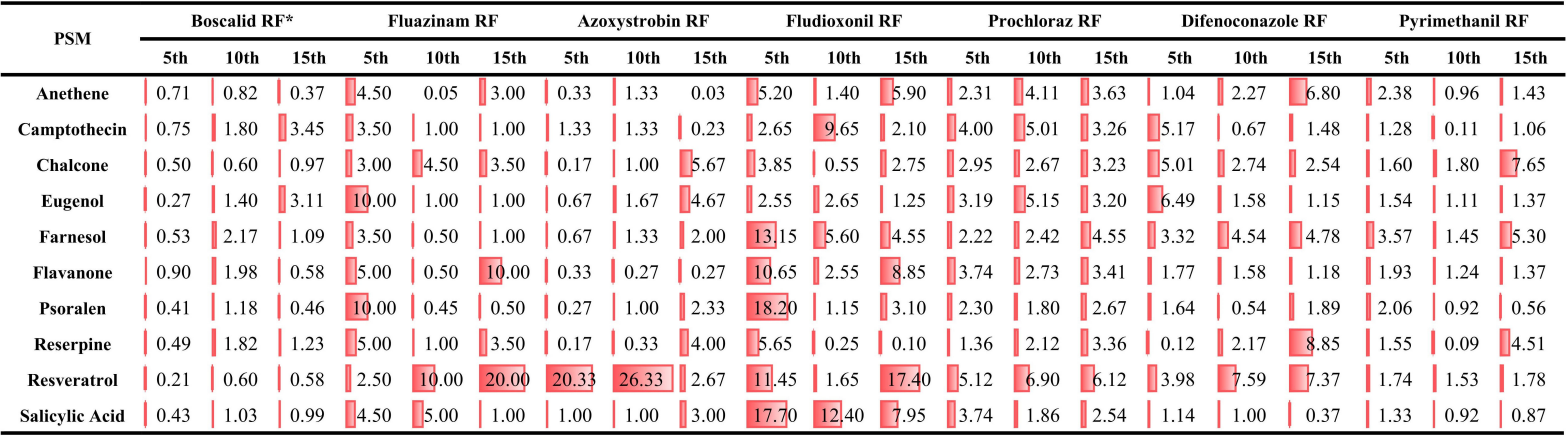
Resistance factors (RFs) ^*^ of *Botrytis cinerea* B05.10 mutants, induced by plant secondary metabolites (PSMs) examined against boscalid, fluazinam, azoxystrobin, fludioxonil, prochloraz, difenoconazole, and pyrimethanil at the 5th, 10th and 15th generations.^*^ RF = effective concentration for 50% growth inhibition (EC_50_) of the fungicide-resistant mutant divided by the EC_50_ of the parental strain.

The parent and the 5th, 10th, and the 15th generations of PSM-adapted *B. cinerea* were assayed for their sensitivities to fungicides (Table S1) and resistance factors (Fig. 2). The sensitivity of all PSM-adapted strains decreased to at least two fungicides. For instance, for *B. cinerea* grown on PDA amended with either eugenol, farnesol, or camptothecin, the EC_50_ to boscalid continuously increased, indicating a reduced sensitivity. The fluazinam sensitivity of *B. cinerea* decreased when treated with either salicylic acid, chalcone, or resveratrol. There were some exemptions. The azoxystrobin sensitivity increased in *B. cinerea* treated with resveratrol in the third generation. With eugenol, farnesol, chalcone, and psoralen treatments, the sensitivity slightly increased in the first generation. The fludioxonil and prochloraz sensitivity had significant decreases in *B. cinerea* treated with most PSMs. The difenoconazole sensitivity decreased when treated with anethene, reserpine, and resveratrol. Chalcone and farnesol treatment decreased the pyrimethanil sensitivity. Overall, the ten PSMs enhanced *B. cinerea* resistance to fungicides.

After 10 transfers on PSM-free PDA, the resistance factors (RFs) of some *B. cinerea* mutants had changed more or less as indicated by factor of sensitivity change (FSC) values. FSC values of the mutants ranged from 0.03 to 11.96 (Table S2). Most of the mutants’ resistance to azoxystrobin, fludioxonil, and pyrimethanil was much higher after 10 transfers. On the other hand, most of the mutants lost their resistance to boscalid, prochloraz, and difenoconazole.

### Transcriptome analysis

**Fig 3:**
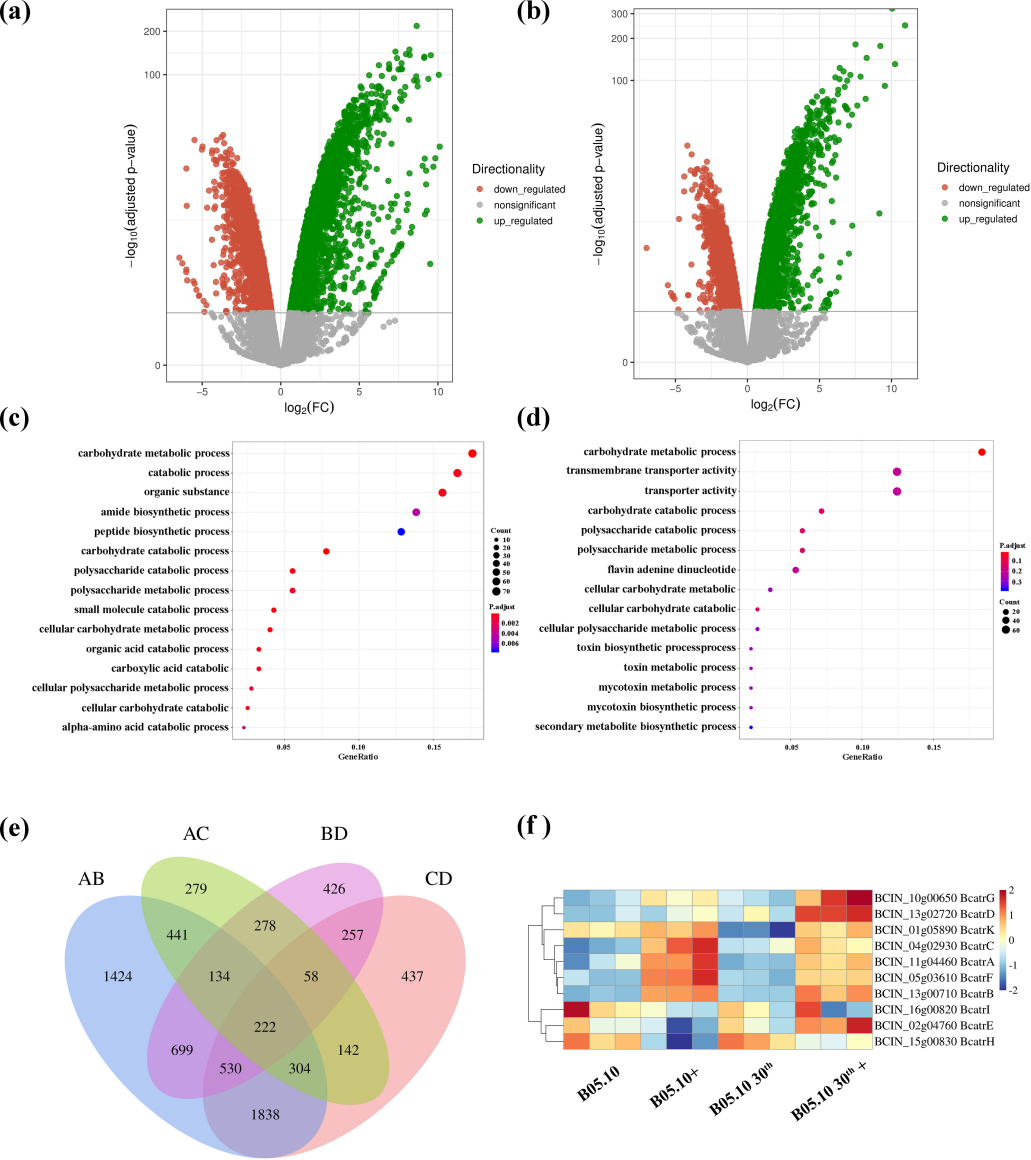
(**a and **b) Volcano plots of differentially expressed genes in azoxystrobin-treated B05.10 compared to non-treated B05.10, with up-regulated genes represented by green dots and down-regulated genes by red dots. (c) Top 15 GO-terms identified in B05.10 exposed to azoxystrobin compared to non-exposed B05.10. (d) Top 15 GO-terms identified in resveratrol-treated B05.10 exposed to azoxystrobin compared to non-exposed resveratrol-treated B05.10. (e) Venn diagram of the number of common and specific differentially expressed genes. AB: group B (azoxystrobin-treated B05.10, 30th generation resveratrol-induced mutant) compared to group A (B05.10); AC: group C (30th generation resveratrol-induced B05.10) compared to group A; BD: group D (azoxystrobin-treated 30th generation resveratrol-induced B05.10) compared to group B; CD: group D compared to group C. (f) Expression profiles of ABC transporter genes in groups A, B, C, and D. The expression is shown as the log2-fold change (Log_2_FC).

**Fig 4:**
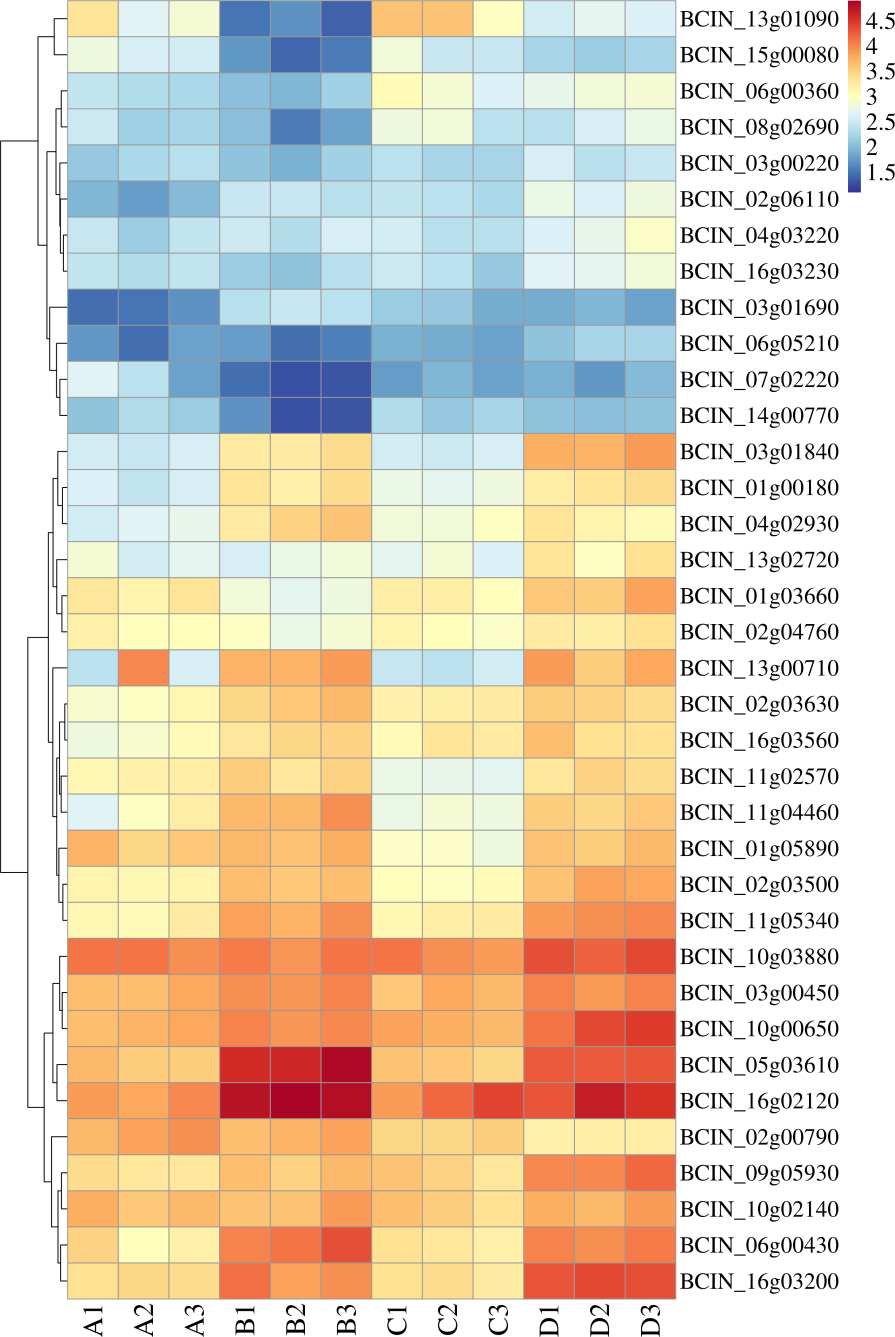
Heat map showing the expression of all ABC transporter genes and related transcription factors in four groups: A (B05.10), B (azoxystrobin-treated B05.10), C (the 30th generation of the B05.10 mutant induced by resveratrol), and D (azoxystrobin-treated B05.10 induced by resveratrol at the 30th generation). Each group has three replicates.

To elucidate the mechanisms involved in the resistance phenotype observed in *B. cinerea* B05.10, a 30th generation resveratrol-induced strain exhibiting an MDR phenotype (Table S3) and a strain with wild-type sensitivity were randomly selected for the transcriptomic analysis through RNA-seq. To identify genes related to resistance, we screened for ABC transporters, MFS transporters, P450, and transcription factors among the differentially expressed genes in group D (the 30th generation of resveratrol-induced B05.10 treated by azoxystrobin) compared to group C (the 30th generation of resveratrol-induced B05.10) (Table 1). Volcano plots were generated to visualize the significantly differentially expressed features (Fig. 3a and b). Gene Ontology (GO) analysis categorized the differential genes in 15 functional groups, with “transmembrane transporter activity” (GO: 0022857) and “transporter activity” (GO: 0005215) being enriched in D vs C (Fig. 3d), but not in group B (azoxystrobin- and azoxystrobin-treated B05.10) vs group A (B05.10) (Fig. 3c). Venn diagram analysis showed common and unique differential metabolites in the four comparisons (AB, AC, BD, and CD) to highlight the effects of resveratrol and azoxystrobin on *B. cinerea* (Fig. 3e). We also examined the expression of ten ABC transporter genes that were shown (Fig. 3f) and identified candidate transcription factors from the reference that were clustered with ABC transporter genes (Fig. 4). AC and BD differential genes include 149 and 219 up-regulation transcription factors coupled with 318 and 237 down-regulation transcription factors, respectively. Among these, eleven transcription factors (BCIN_01g03660, BCIN_03g01840, BCIN_03g06600, BCIN_06g05210, BCIN_09g05540, BCIN_10g03530, BCIN_13g03910, BCIN_13g05610, BCIN_14g00770, BCIN_15g00080, and BCIN_16g03230) were found to be up-regulated in the resveratrol-induced strain compared to the non-induced strain selected from the reference, which was related to drug resistance in microorganisms including Tac1p, Mrr1, and Mrr2 in *Candida albicans* (25–27) (Table S4). On the contrary, BCIN_02g01900, BCIN_03g02160, and BCIN_13g02900 were found to be down-regulated. Meanwhile, 13g04870, BCIN_13g03910, BCIN_03g00220, BCIN_03g00450, BCIN_03g06600, BCIN_06g05210, BCIN_10g03880, BCIN_04g03220, BCIN_16g03230, BCIN_15g00120, BCIN_01g03660, BCIN_02g01900, BCIN_02g03500, BCIN_11g05340, BCIN_16g03200, and BCIN_03g01840 were up-regulated in group D compared to group C.

**TABLE 1:** Differential genes in the transcriptome of resveratrol-induced B05.10 and B05.10 strain^*a*^

	Differential gene (up-regulation）				Differential gene (down-regulation）			
	AB	CD	AC	BD	AB	CD	AC	BD
ABC transporter	24	20	7	9	10	2	4	7
MFS	103	85	35	45	59	43	30	46
P450	103	83	42	48	13	140	43	67
Transcription factor	556	422	149	219	558	318	118	237

^*a*^ AB: group B (azoxystrobin-treated B05.10 mutant ) compared to group A (B05.10); CD: group D (azoxystrobin-treated B05.10 induced by resveratrol at the 30th generation) compared to group C (the 30th generation of resveratrol-induced B05.10); AC: group C compared to group A, BD: group D compared to group B.

**Fig 5:**
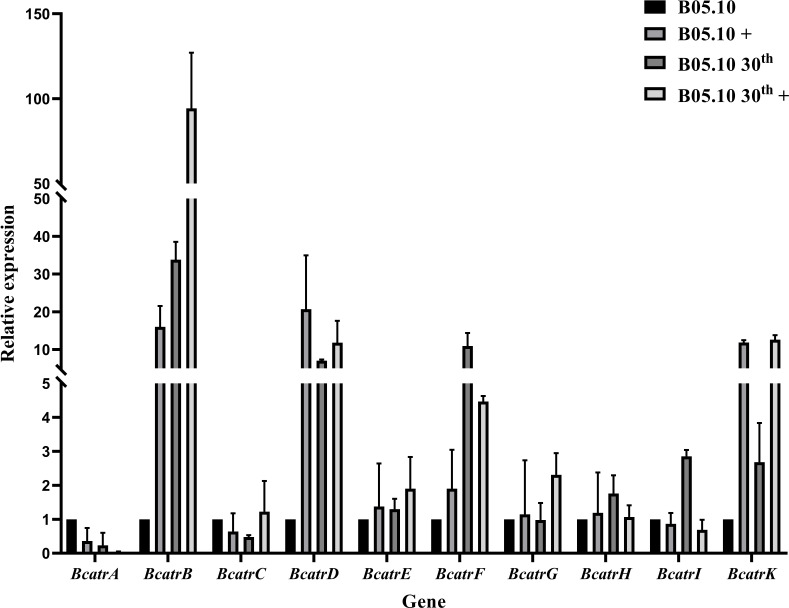
Quantitative polymerase chain reaction (qPCR) analysis of 12 ABC transporters genes in non-treated and azoxystrobin-treated B05.10 strain. + indicates azoxystrobin added, and 30th indicates the resveratrol-induced mutant at the 30th generation.

The RNA-seq results revealed an overexpression of ABC transporter genes in the groups at the 30th generation. To validate these findings, we performed qPCR assays on RNA samples obtained both before and after 4-h azoxystrobin treatment, measuring the expression levels of 10 ABC transporter genes (Fig. 5). Our results showed that overall, the qRT-PCR results were mostly consistent with the RNA-seq data, although there were some minor variations in fold-change between the RNA-seq and qRT-PCR results for some genes. These findings affirm the reliability of our experimental results. Additionally, *BcatrB*, *BcatrD,* and *BcatrK* were identified as highly upregulated genes following azoxystrobin treatment, as confirmed by qPCR. The transcription factors associated with ABC transporter genes were screened through expression profiles.

### ABC gene expression of the PSM-adapted mutant of *Botrytis cinerea*

**Fig 6:**
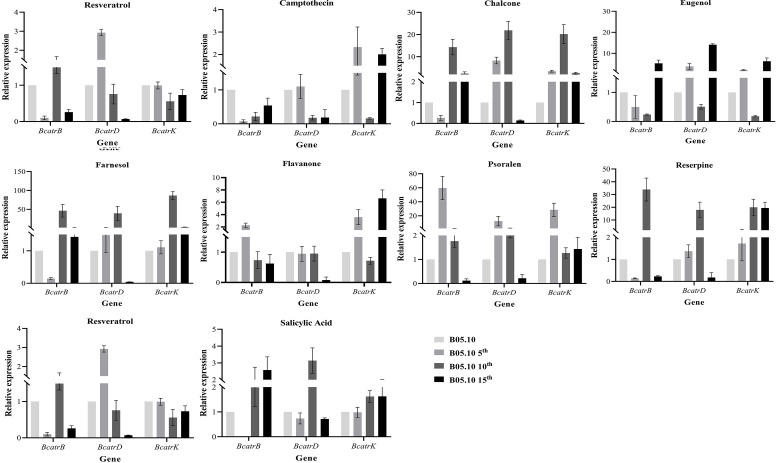
Expression of ABC transporter genes *BcatrB, BcatrD,* and *BcatrK* of *Botrytis cinerea* B05.10 and its 5th (B05.10 5th), 10th (B05.10 10th), and 15th (B05.10 15th) transfers on potato dextrose agar amended with plant secondary metabolites.

The expression of ABC transporter genes *BcatrB*, *BcatrD,* and *BcatrK* was measured through quantitative PCR (qPCR) in the parents and the 5th, 10th, and the 15th generations of *B. cinerea* B05.10 co-cultured with one of the ten PSMs. The expression levels of *BcatrB, BcatrD,* and *BcatrK* in the three generations of *B. cinerea* were all up-regulated when exposed to salicylic acid, reserpine, chalcone, eugenol, farnesol, and anethene domestication (Fig. 6). PSMs such as resveratrol and flavanone induced the overexpression of one or two of the three ABC transporter genes. Most PSMs stimulated the overexpression of the transporter genes.

### Contribution of *BcatrB*, *BcatrD,* and *BcatrK* overexpression to multidrug resistance in *Botrytis cinerea*

**Fig 7:**
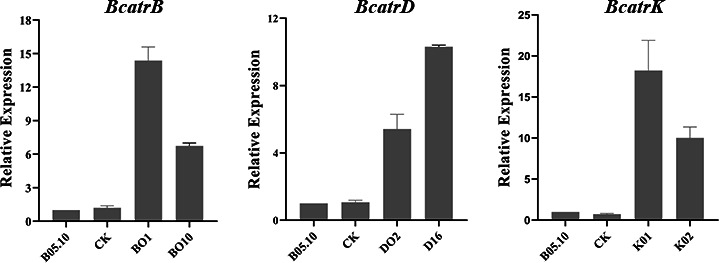
*BcatrB, BcatrD,* and *BcatrK* expressions in the transformants for B05.10 with the empty vector (CK), *BcatrB* (BO1 and BO10), *BcatrD* (DO2 and DO16), and *BcatrK* (KO1 and KO2) determined using quantitative polymerase chain reaction (qPCR). Data are presented as mean values ± standard deviation (SD) from three independent replicates

A pxEH-GFP-plasmid was constructed for overexpression. PCR results indicated the plasmids were successfully transformed into B05.10. The expression of *BcatrB, BcatrD,* and *BcatrK* in the transformants was 8 to 18 folds higher than that of the wild-type B05.10 (Fig. 7), indicating overexpression.

### Sensitivity of *BcatrB*, *BcatrD,* and *BcatrK* overexpression transformants to fungicides

**Fig 8:**
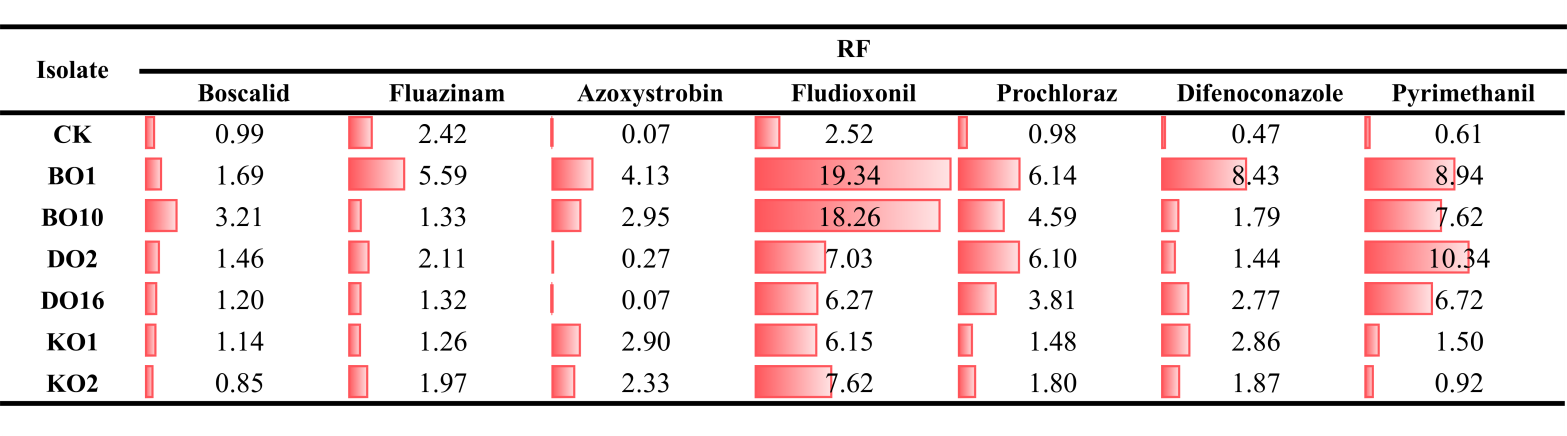
Resistance factors (RFs) ^*^ of *Botrytis cinerea* mutants examined against boscalid, fluazinam, azoxystrobin, fludioxonil, prochloraz, difenoconazole, and pyrimethanil.^*^ RF = effective concentration for 50% growth inhibition (EC_50_) of the fungicide-resistant mutant divided by the EC_50_ of the parental strain CK: the transformants for B05.10 with the empty vector; BO1 and BO10: the transformants for B05.10 with *BcatrB*; DO2 and DO16: the transformants for B05.10 with *BcatrD*; KO1 and KO2: the transformants for B05.10 with *BcatrK*.

The sensitivities of *B. cinerea* transformants to fungicides were determined (Fig. 8; Table S5). The pxEH-GFP-plasmid transformant was a control (CK), which had no significant difference compared with the wild-type B05.10 strain. *BcatrB* overexpression in transformants BO1 and BO10 resulted in a significant increase in EC_50_ values of seven fungicides, particularly notable for fludioxonil and pyrimethanil, with RF values ranging from 7.6 to 19.3, respectively. Meanwhile, *BcatrD* overexpression in transformants DO2 and DO16 led to increased EC_50_ values of fludioxonil, difenoconazole, prochloraz, and pyrimethanil. Lastly, *BcatrK* overexpression in transformants KO1 and KO2 resulted in a slight increase in EC_50_ values for azoxystrobin, fludioxonil, and difenoconazole, with RF values ranging from 1.9 to 6.2. These results suggested the sensitivity of *B. cinerea* to fungicides was associated with ABC transporters *BcatrB*, *BcatrD,* and *BcatrK*.

## DISCUSSION

In this study, we have demonstrated that PSMs including eugenol, farnesol, reserpine, and resveratrol elicited the overexpression of ABC transporter genes, including *BcatrB*, *BcatrD,* and *BcatrK,* in *B. cinerea*. As a result of continued PSM exposure over multiple generations, the response of the fungus was adaptive to both PSM stresses and fungicides. Therefore, it was proved that continuous exposure to PSMs can induce a progressive and elevated adaptability to PSMs, which helps *B. cinerea* to be resistant to multiple fungicides.

This type of preadaptation has been observed in other organisms, including insects and bacteria (23, 28–32). For instance, flavonoids induce the overexpression of the P450 gene in whitefly (*Bemisia tabaci*), leading to metabolic detoxification and resistance to insecticides thiamethoxam and flufenoxuron (23). Similarly, quercetin induces the expresson of P450 and carboxylesterase, leading to lambda-cyhalothrin resistance in cotton bollworm (*Helicoverpa armigera*) (29, 33). Moreover, the secondary metabolites from *Pseudomonas aeruginosa*, such as phenazine 1-carboxylic acid, phenazine 1-carboxamide, paerucumarin, indole, salicylic acid, prothiocyanide, ergothioneine, polyamines, H_2_S, and *Pseudomonas* quinolone signal can improve the antibiotic resistance of medical pathogens (10). This preadaptation is primarily due to the enhanced efflux (34, 35). Otherwise, capsidiol induces high expression of *Bccpdh,* which encodes a dehydrogenase in *B. cinerea*. This gene has been predicted to result in decreased sensitivity of *B. cinerea* to capsodiol, and the resistance is related to plant metabolites (36).

The major sources of preadaptation in insects and bacteria are P450 detoxification of secondary metabolites and the efflux system. Similarly, the adaptation of plant pathogens to xenobiotics, which leads to insensitivity to multiple fungicides, is also due to the efflux (10, 37–39), including ABC and MFS efflux transporter genes (2, 40, 41). To explore the main factor responsible for MDR, we performed genetic transformation experiments and found that overexpression of three ABC transporter genes, *BcatrB, BcatrD,* and *BcatrK,* caused resistance to fungicides having different MOAs in *B. cinerea* (Fig. 8). This finding is consistent with the previous report (8, 10, 42). PSM-induced overexpression of three ABC genes contributes to multidrug resistance. Moreover, we observed significant overexpression of these three ABC transporter genes in PSM-induced strains, regardless of the presence or absence of PSM stimulation, after 15 generations of culture on PDA (the latter data not shown in this study).

We hypothesize that MDR that occurred in the field may be stimulated by PSMs. This hypothesis is supported by other reports (10, 11, 14), which have demonstrated that PSMs can affect ABC efflux proteins. When exposed to PSMs during growth, the sensitivity of *B. cinerea* to the PSMs decreased, accompanied with the up-regulation of the three ABC efflux genes. It is acknowledged that under experimental conditions, the concentration of some secondary metabolites may be much higher than that in plants. The purpose of having a higher concentration is to create a high screening pressure, allowing the fungus to adapt to the stress in a shorter period. However, in an *in vivo* test using a living leaf, the selection pressure may be different compared with *in vitro* conditions due to differences in nutritional conditions and various types of PSMs. The presence of stress helps the pathogen adapt to the fungicide pressure and more likely develop MDR. Therefore, PSMs impacted the pathogen during plant–pathogen interactions and contribute to the development of MDR.

Fungicide resistance is usually determined by genetic variation and mutation (43). In fungi, whether these two concepts reflect genetic or non-genetic change of resistance of pathogens deserves discussion. Non-genetic variation could be a stress response, and we have used the term “mutant” to describe adaptative generations of the fungus in this study. Similarly, bacteria have been shown to develop multiple phenotypic variants in response to various selective pressures, such as immune / defense challenges, antimicrobial therapy, and oxygen limitation (44, 45). In this study, salicylic acid-induced mutants had reduced susceptibility to PSM, as well as boscalid and azoxystrobin. The stable inheritance after 10 generations of relay culture on a drug-free medium was also found to have significant up-regulation of *BcatrB* and *BcatrK*. In addition, similar results were found in flavanone-induced mutants exposed to fludioxonil through overexpression of *BcatrK*. However, the exact mechanism of resistance induction by PSMs, whether through stably or unstably inherited changes in the pathogen, remains unclarified. This is an interesting and important issue that is worthy of further investigation. While various fungicides were intentionally selected to represent different MOAs, the observed efflux effect of ABC transporter proteins may be related to the chemical structures of the fungicides or PSMs. It is plausible that different fungicides may engage distinct ABC transporters in efflux mechanisms that contribute to MDR, which needs further in-depth investigations.

It is an interesting phenomenon that the sensitivity of mutants against PSMs and fungicides has a regression at the 15th generation. In the context of secondary metabolite treatment, organisms may survive in a responsive status when encountering stresses, but the long-term maintenance of this status is detrimental to the normal development of the organism, which is called resistance cost in resistant strains (46). That is a reason some mechanisms will appear in the organism that can regulate this stress state, preventing the organism from remaining in this state for a long time to promote better growth. This might explain why the 15th generation is more sensitive compared to the 10th generation. It was reported that nematodes also exhibit reduced resistance under the stress of antimycin and show the same trend of stress tolerance as our results (increased from the F1 to F3 progeny but decreased in the F4 progeny), and these phenotypes may be related to the properties and applied dosage of the drug (47).

The biochemical or genetic propensity for resistance is the base on which the selection pressure operates, and host characteristics and movement are key ecological traits that influence effective selection intensities for resistance (48). Host–pathogen interactions continuously compel species to adapt to each other, making them one of the most complex and fascinating models of the evolutionary interplay between organisms (49). Resveratrol, which is obtained from the host plant, induces intracellular signal transduction pathways, which ultimately lead to changes in the gene expression patterns of the cells by activating the stimulus-responsive transcription factors such as CREB, AP-1, Egr-1, Elk-1, and Nrf2 (50). Farnesol also activated transcription factors Tac1 and Znc1 to up-regulate the ABC transporter *CDR1*, which was crucial in drug resistance (51, 52). Following activation, these transcription factors induce transcription of delayed response genes. *Botrytis cinerea* MDR1 strains showed higher expression of the ABC transporter *BcatrB* due to transcription regulation by transcription factor Mrr1 (2). In this research, based on transcriptome data analysis, the induction of plant secondary metabolites, such as resveratrol, can lead to overexpression of ABC transporter genes, which may be contributed to the effect of secondary metabolites on transcription factors in *B. cinerea*. Moreover, it has been reported that compounds such as pyocyanin (PYO) in *Pseudomonas aeruginosa* and indole in *Escherichia coli*, which were structurally similar to antibiotics like ciprofloxacin and chloramphenicol, respectively, contribute to preadaptation via efflux pumps (38, 53). It also found that some fungicides and examined secondary metabolites showed complex and abundant chemical structure, including benzene rings, aldehyde, alkene, and oxhydryl in this study. It is inferred that PSMs with structural similarities of fungicides may regulate up-regulation of ABC transporters via responding of transcriptional factors or dircted combination. Candidate transcription factors relative to ABC transporter gene expression were screened using fold change of expression and cluster analysis and will be validated in further studies. The results of this work can be a good reference for studying other plant pathogens.

## MATERIALS AND METHODS

### Chemicals

Technical-grade resveratrol (a.i. 99%), psoralen (98%), camptothecin (97%), reserpine (98%), and eugenol (99%) were purchased from Shanghai Maclean Biochemical Technology Co., LTD (Shanghai, China). Salicylic acid was provided by Beijing Solarbio Technology Co., LTD. Chalcone (97%) and anethene (99%) were obtained from Biochemical Technology Co., Ltd. (Shanghai, China). Flavanones (98%) and farnesol (mixture of isomers) (SG 0.89) were obtained from Tokyo Chemical Industry Co., Ltd. (Tokyo, Japan). The fungicides and inhibitor of the alternative respiratory pathway used were azoxystrobin (98.0%, Syngenta Biotechnology, Shanghai), salicylhydroxamic acid (SHAM) (99%, Shanghai Marklin Biochemistry, China), boscalid (99%, BASF Corporation, Nanjing, China), fluazinam (98.4%, Ishihara Sangyo Kaisha Ltd., Japan), fludioxonil (97.9%, Syngenta Biotechnology, Co., Ltd, China), difenoconazole (98.4%, Yulong Chemical Industrial Co., Ltd., Hangzhou), pyrimethanil (93%, Jiangsu Limin Chemical Industrial Co., Ltd., China), and prochloraz (98%, Jiangsu Huifeng Agrochemical Co., Ltd., Dafeng, China), and all PSMs were dissolved in dimethyl sulfoxide (DMSO) to prepare a stock solution (1 × 10^5^ µg/mL) and kept in darkness at −20°C.

### Pathogen and growth conditions

**Fig 9:**
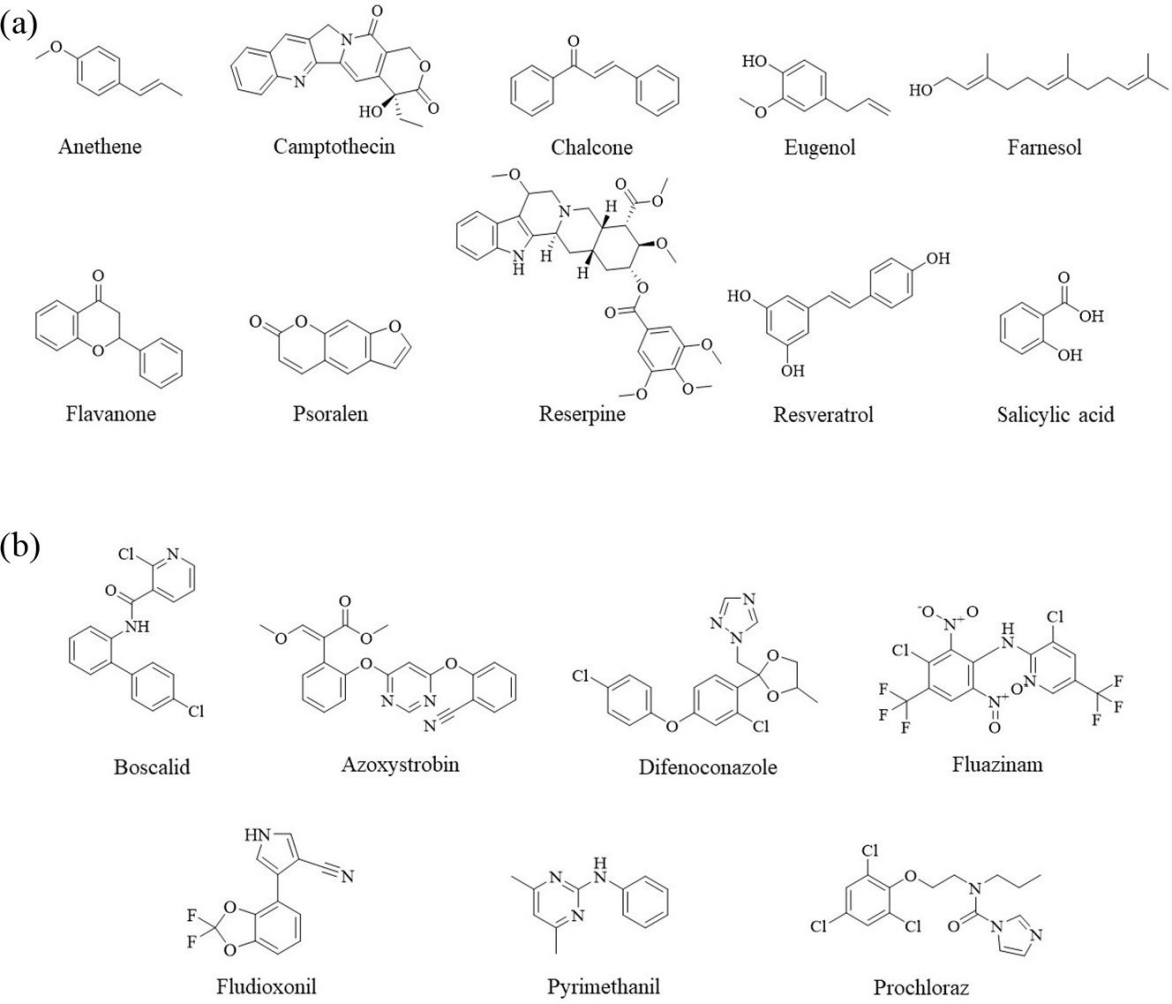
Chemical structures of plant secondary metabolites (**a**) and fungicides (**b**) used in this study.

*Botrytis cinerea* B05.10 was used as a standard strain. Mycelial plugs (5 mm diameter) of *B. cinerea* strains were cultivated on potato dextrose agar (PDA) at 18°C in the dark for 3 to 5 days. To produce a conidial suspension, the strains were cultured in the dark at 18°C on carrot agar (CA) for 3 to 5 days and placed under a black light lamp for 5 days. Conidia were washed with sterile water and filtered through three layers of filter paper to eliminate mycelia. Ten PSMs, namely, resveratrol, reserpine, chalcone, flavanone, eugenol, farnesol, anethene, camptothecin, salicylic acid, and psoralen (Fig. 9a), were used to induce *B. cinerea* for preadaptation at different concentrations (Table 2). The 5th, 10th, and 15th generation strains exposed to PSMs were collected and used for further testing.

**TABLE 2:** Concentrations of plant secondary metabolites (PSMs) amended in potato dextrose agar for growing *Botrytis cinerea* B05.10

PSM	Concentration (µg/mL)
Anethene	100
Camptothecin	100
Chalcone	50
Eugenol	50
Farnesol	100
Flavanone	25
Psoralen	10
Reserpine	50
Resveratrol	100
Salicylic acid	100

### Effects of PSMs on *Botrytis cinerea* growth

The sensitivities of *B. cinerea* were examined by following Song et al.’s method (54) with some modifications. Briefly, PDA was amended with one of the ten PSMs at final concentrations of 10, 25, 50, and 100 mg/L with three replicates. Mycelial growth of *B. cinerea* under the PSM treatment was measured for the 5th, 10th, and 15th generations.

### Characterization of *Botrytis cinerea* induced by PSMs

Fungicides (Fig. 9b) were dissolved using DMSO to form a 10^5^ mg/L concentration stock solution, which was further diluted into a series of six concentrations as working solutions, namely, boscalid (0.1, 0.5, 1, 5, and 10 mg/L), azoxystrobin (0.1, 0.5, 1, 5, and 10 mg/L), fluazinam (0.01, 0.05, 0.1, 0.5, and 1 mg/L), fludioxonil (0.01, 0.05, 0.1, 0.5, and 1 mg/L), difenoconazole (0.01, 0.05, 0.1, 0.5, and 1 mg/L), pyrimethanil (0.1, 0.25, 0.5, 1, and 2.5 mg/L), and prochloraz (0.01, 0.05, 0.1, 0.5, and 1 mg/L). The fungicide-free DMSO solution was used as a control. All concentrations were prepared in triplicate.

Mycelial growth of the 5th, 10th, and 15th generations of PSM-treated *B. cinerea* was measured under various concentrations of fungicides, and EC_50_ was calculated (55). RF was calculated as RF = EC_50_ of the mutant / EC_50_ of the parental strain. To assess the stability of the resistant mutants, mycelial plugs of ten 15th generation-induced mutants were subjected to 10 successive culture transfers on fungicide-free PDA plates, and EC_50_ values were measured on the 1st and 10th transfers. The stability of resistance was calculated by the FSC, where FSC was calculated by dividing the RF of the tenth subculture by that of the first (56).

### Transcriptome sequencing and qRT-PCR

The sensitivities of the 20th and 30th generations of resveratrol-induced mutants were examined against seven fungicides as described in preceding section. RNA was extracted from four groups of *B. cinerea* B05.10: group A (B05.10), group B (azoxystrobin-treated B05.10 mutant induced by resveratrol at the 30th generation), group C (the 30th generation of resveratrol-induced B05.10 mutant), and group D (azoxystrobin-treated B05.10 mutant induced by azoxystrobin). The extraction was carried out using the NEBNext Ultra RNA Library Prep Kit for Illumina (NEB, Ipswich, MA, United States). Sequencing was performed on AMPure XP beads as 250–300 bp paired-end reads and mapped using the *B. cinerea* B05.10 reference genome (https://www.ncbi.nlm.nih.gov/data-hub/genome/GCF_000143535.2/). The Qubit 2.0 fluorometer was used for preliminary quantification, and the insert size of the library was detected by using an Agilent 2100 bioanalyzer (Agilent Technologies, CA, USA) following library construction. The effective concentration of the library was accurately quantified by qRT-PCR, and the differential genes were screened by |log_2_(FoldChange)|> 1 and *P* < 0.05 as the cut-off criteria. Three independent biological replicates were analyzed in this experiment.

The most significant 15 terms were selected to draw a scatter plot for display in GO enrichment analysis. The volcano plot can visually display the distribution of differentially expressed genes for comparison of each combination.

RNA extraction was performed as described previously. The PrimeScript RT reagent Kit with gDNA Eraser (Takara, Beijing, China) was used to synthesize cDNAs. Ten ABC transporter genes were validated by the transcriptome results by qPCR (*BcatrA* BCIN_11g04460, *BcatrB* BCIN_13g00710, *BcatrC* BCIN_04g02930, *BcatrD* BCIN_13g02720, *BcatrE* BCIN_02g04760, *BcatrF* BCIN_05g03610, *BcatrG* BCIN_10g00650, *BcatrH* BCIN_15g00830, *BcatrI* BCIN_16g00820, and *BcatrK* BCIN_01g05890). qPCR was performed on an ABI7500 sequence detection system (Applied Biosystems, California, United States) using the FastSYBR Mixture kit (Beijing ComWin Biotech Co., Ltd., Beijing, China) (Table S6) and the cDNA as a template. In the qPCR analysis, the reaction system was mixed with 10 µL 2 × FastSYBR, 0.4/0.4 µL mixture forward/reverse primer, 1 µL template cDNA, and 8.2 µL ddH_2_O. The settings of the thermocycler included denaturation at 95°C for 2 min, followed by 40 cycles of 95°C for 10 s, and 60°C for 34 s. The relative expression of genes was calculated using the 2^−ΔΔCt^ method (57), and the actin genes (BCIN_16g02020) were used as a reference to normalize the quantification of the ABC gene expression levels. The experiment was conducted twice, and each treatment had three replicates.

### qPCR of *Botrytis cinerea* induced by PSMs

Test cultures of gene quantification included *B. cinerea* B05.10 and its 5th, 10th, and 15th generations shaken at 180 rpm at 18°C under PSM treatments. Biological materials of induced *B. cinerea* in potato dextrose broth (PDB) were collected in an RNA-free tube. Total RNA was extracted from the samples, and qPCR was performed as described previously.

### Construction of the gene expression cassette

The pxEH-GFP plasmid was constructed by inserting the OliC promoter and GFP tag into the pxEH vector (58) using BamHI and HindIII restriction sites, which served as an overexpression vector. The ABC transporter genes *BcatrB, BcatrD,* and *BcatrK* were amplified from B05.10 cDNA using polymerase chain reaction (PCR) with SalI and XbaI restriction enzymes. PCR was performed in a reaction of 20 µL mixture of EasyTaq DNA Polymerase (TransGen, Beijing, China). Thermal cycler settings included an initial denaturation at 94°C for 5 min, followed by 35 cycles of denaturation at 94°C for 30 s, annealing at 61°C for 30 s, and extension at 72°C for 2 min, which was ended with an extension at 72°C for 10 min. The PCR products were retrieved and purified using the Gel Extraction Kit (CWBIO, Beijing, China).

### Gene transformation in *Botrytis cinerea*

The pxEH-GFP plasmid and expression vectors were transformed into *Agrobacterium tumefaciens* strain AGL-1 and then transformed into *B. cinerea B05.10* spores. Transformants were successfully screened using hygromycin phosphotransferase gene (HPH) resistance methods (58) and identified through PCR (Table S6). The expression of *BcatrB, BcatrD,* and *BcatrK* genes in their transformants was measured using qPCR (Table S6). Biological samples from the transformants grown on PDA containing 100 µg/mL hygromycin were collected in an RNA-free tube. Total RNA was extracted from all samples following the manufacturer’s instructions. The programs of cDNA synthesis and qPCR of three ABC transporter genes in respectively their over-expression mutants were the same as those in preceding section.

### Fungicide sensitivities of *Botrytis cinerea* transformants

Fungicides were dissolved in dimethyl sulfoxide (DMSO). The *B. cinerea* transformants were grown on PDA amended with seven fungicides, and EC_50_ was calculated as described in preceding section. The inhibition rates were calculated by comparing the growth of *B. cinerea* transformants on fungicide-amended and to non-amended (control) PDA. To keep the growth conditions consistent, the final concentration of DMSO in PDA was kept at 0.1% (v/v). Each experiment was performed with three replicate plates, and the experiment was conducted twice.

### Statistical analysis

Data were analyzed using GraphPad Prism 8.4.3 (GraphPad Software Inc., San Diego, CA, USA). Significance differences of treatments were analyzed by the one-way ANOVA analysis (*α* = 0.05).

## SUPPLEMENTAL MATERIAL

Supplemental material mbio.02237-23-s0001.xlsx Tables S1 to S6.

ASM does not own the copyrights to Supplemental Material that may be linked to, or accessed through, an article. The authors have granted ASM a non-exclusive, world-wide license to publish the Supplemental Material files. Please contact the corresponding author directly for reuse.

## References

[1] Pommer EH, Lorenz G. 1982. Resistance of Botrytis cinerea Pers. to dicarboximide fungicides-a literature review. Crop Protection 1:221–230. doi:10.1016/0261-2194(82)90044-8

[2] Kretschmer M, Leroch M, Mosbach A, Walker AS, Fillinger S, Mernke D, Schoonbeek HJ, Pradier JM, Leroux P, De Waard MA, Hahn M. 2009. Fungicide-driven evolution and molecular basis of multidrug resistance in field populations of the grey mold fungus Botrytis cinerea. PLoS Pathog 5:e1000696. doi:10.1371/journal.ppat.1000696 20019793 PMC2785876

[3] Cheng XK, Dai T, Hu ZH, Cui TS, Wang WZ, Han P, Hu ML, Hao JJ, Liu PF, Liu XL. 2022. Cytochrome P450 and glutathione S-transferase confer metabolic resistance to SYP-14288 and multi-drug resistance in Rhizoctonia solani. Front Microbiol 13:806339. doi:10.3389/fmicb.2022.806339 35387083 PMC8977892

[4] Hayashi K. 2003. ABC and MFS transporters from Botrytis cinerea involved in sensitivity to fungicides and natural toxic compounds. Wageningen University and Research.

[5] Higgins CF. 1992. ABC transporters: from microorganisms to man. Annu Rev Cell Biol 8:67–113. doi:10.1146/annurev.cb.08.110192.000435 1282354

[6] Stergiopoulos I, Zwiers L-H, De Waard MA. 2002. Secretion of natural and synthetic toxic compounds from filamentous fungi by membrane transporters of the ATP-binding cassette and major facilitator superfamily. Eur J Plant Pathol 108:719–734. doi:10.1023/A:1020604716500

[7] Vermeulen T, Schoonbeek H, De Waard MA. 2001. The ABC transporter BcatrB from Botrytis cinerea is a determinant of the activity of the phenylpyrrole fungicide fludioxonil. Pest Manag Sci 57:393–402. doi:10.1002/ps.309 11374155

[8] Pane C, Rekab D, Firrao G, Ruocco M, Scala F. 2008. A novel gene coding for an ABC transporter in Boterytis cinerea (Botryotinia fuckeliana) is involved in resistance to H_2_O_2_. Can J Plant Pathol 90:453–462. doi:10.4454/jpp.v90i3.687

[9] Rupp S, Plesken C, Rumsey S, Dowling M, Schnabel G, Weber RWS, Hahn M. 2017. Botrytis fragariae, a new species causing gray mold on strawberries, shows high frequencies of specific and efflux-based fungicide resistance. Appl Environ Microbiol 83:e00269-17. doi:10.1128/AEM.00269-17 28235878 PMC5394320

[10] Jeandet P, Delaunois B, Conreux A, Donnez D, Nuzzo V, Cordelier S, Clément C, Courot E. 2010. Biosynthesis, metabolism, molecular engineering, and biological functions of stilbene phytoalexins in plants. Biofactors 36:331–341. doi:10.1002/biof.108 20726013

[11] Schoonbeek H, Del Sorbo G, De Waard MA. 2001. The ABC transporter BcatrB affects the sensitivity of Botrytis cinerea to the phytoalexin resveratrol and the fungicide fenpiclonil. Mol Plant Microbe Interact 14:562–571. doi:10.1094/MPMI.2001.14.4.562 11310744

[12] Nakaune R, Hamamoto H, Imada J, Akutsu K, Hibi T. 2002. A novel ABC transporter gene, PMR5, is involved in multidrug resistance in the phytopathogenic fungus Penicillium digitatum. Mol Genet Genomics 267:179–185. doi:10.1007/s00438-002-0649-6 11976961

[13] Hayashi K, Schoonbeek HJ, De Waard MA. 2002. Bcmfs1, a novel major facilitator superfamily transporter from Botrytis cinerea, provides tolerance towards the natural toxic compounds camptothecin and cercosporin and towards fungicides. Appl Environ Microbiol 68:4996–5004. doi:10.1128/AEM.68.10.4996-5004.2002 12324349 PMC126426

[14] Del Sorbo G, Andrade AC, Van Nistelrooy JG, Van Kan JA, Balzi E, De Waard MA. 1997. Multidrug resistance in Aspergillus nidulans involves novel ATP-binding cassette transporters. Mol Gen Genet 254:417–426. doi:10.1007/s004380050434 9180695

[15] De Waard MA, Andrade AC, Hayashi K, Schoonbeek HJ, Stergiopoulos I, Zwiers LH. 2006. Impact of fungal drug transporters on fungicide sensitivity, multidrug resistance and virulence. Pest Manag Sci 62:195–207. doi:10.1002/ps.1150 16475240

[16] Gyémánt N, Tanaka M, Antus S, Hohmann J, Csuka O, Mándoky L, Molnár J. 2005. In vitro search for synergy between flavonoids and epirubicin on multidrug-resistant cancer cells. In Vivo 19:367–374. 15796199

[17] Togashi N, Hamashima H, Shiraishi A, Inoue Y, Takano A. 2010. Antibacterial activities against Staphylococcus aureus of terpene alcohols with aliphatic carbon chains. Journal of Essential Oil Research 22:263–269. doi:10.1080/10412905.2010.9700321

[18] Tong Y, Liu M, Zhang Y, Liu X, Huang R, Song F, Dai H, Ren B, Sun N, Pei G, Bian J, Jia XM, Huang G, Zhou X, Li S, Zhang B, Fukuda T, Tomoda H, Ōmura S, Cannon RD, Calderone R, Zhang L. 2016. Beauvericin counteracted multi-drug resistant Candida albicans by blocking ABC transporters. Synth Syst Biotechnol 1:158–168. doi:10.1016/j.synbio.2016.10.001 29062940 PMC5640798

[19] Yoganathan S, Alagaratnam A, Acharekar N, Kong J. 2021. Ellagic acid and schisandrins: natural biaryl polyphenols with therapeutic potential to overcome multidrug resistance in cancer. Cells 10:458. doi:10.3390/cells10020458 33669953 PMC7924821

[20] Ajouz S, Nicot PC, Bardin M. 2010. Adaptation to pyrrolnitrin in Botrytis cinerea and cost of resistance. Plant Pathology 59:556–566. doi:10.1111/j.1365-3059.2009.02230.x

[21] Schoonbeek H, Raaijmakers JM, De Waard MA. 2002. Fungal ABC transporters and microbial interactions in natural environments. Mol Plant Microbe Interact 15:1165–1172. doi:10.1094/MPMI.2002.15.11.1165 12423022

[22] Perry EK, Meirelles LA, Newman DK. 2022. From the soil to the clinic: the impact of microbial secondary metabolites on antibiotic tolerance and resistance. Nat Rev Microbiol 20:129–142. doi:10.1038/s41579-021-00620-w 34531577 PMC8857043

[23] Zhang Q, Yang F, Tong H, Hu Y, Zhang X, Tian T, Zhang Y, Su Q. 2021. Plant flavonoids enhance the tolerance to thiamethoxam and flupyradifurone in whitefly Bemisia tabaci (Hemiptera: Aleyrodidae). Pestic Biochem Physiol 171:104744. doi:10.1016/j.pestbp.2020.104744 33357566

[24] Wu ZC, Gao TQ, Liang ZY, Hao JJ, Liu PF, Liu XL. 2023. Dynamic changes in plant secondary metabolites induced by Botrytis cinerea infection. Metabolites 13:654. doi:10.3390/metabo13050654 37233695 PMC10223084

[25] Song M, Zhang M, Lu J, Xie F, Song J, Luan X, Hou X, Lou H, Chang W. 2022. Palmarumycin P3 reverses Mrr1-mediated azole resistance by blocking the efflux pump Mdr1. Antimicrob Agents Chemother 66:e0212621. doi:10.1128/aac.02126-21 35041505 PMC8923160

[26] Nishimoto AT, Zhang Q, Hazlett B, Morschhäuser J, Rogers PD. 2019. Contribution of clinically derived mutations in the gene encoding the zinc cluster transcription factor Mrr2 to fluconazole antifungal resistance and CDR1 expression in Candida albicans. Antimicrob Agents Chemother 63:10–1128. doi:10.1128/AAC.00078-19 PMC6496071 30833425

[27] Lohberger A, Coste AT, Sanglard D. 2014. Distinct roles of Candida albicans drug resistance transcription factors TAC1, MRR1, and UPC2 in virulence. Eukaryot Cell 13:127–142. doi:10.1128/EC.00245-13 24243794 PMC3910953

[28] Zhang L, Lv S, Liu Y, Yang L, Liang P, Gao X. 2020. Cellular redox-related transcription factor Nrf2 mediation of HaTrf response to host plant allelochemical 2-tridecanone in Helicoverpa armigera. J. Agric Food Chem 68:6919–6926. doi:10.1021/acs.jafc.0c02080 32463694

[29] Shatalin K, Shatalina E, Mironov A, Nudler E. 2011. H_2_S: a universal defense against antibiotics in bacteria. Science 334:986–990. doi:10.1126/science.1209855 22096201

[30] Hazan R, Que YA, Maura D, Strobel B, Majcherczyk PA, Hopper LR, Wilbur DJ, Hreha TN, Barquera B, Rahme LG. 2016. Auto poisoning of the respiratory chain by a quorum-sensing-regulated molecule favors biofilm formation and antibiotic tolerance. Curr Biol 26:195–206. doi:10.1016/j.cub.2015.11.056 26776731 PMC4729643

[31] Hall JW, Yang J, Guo H, Ji Y. 2017. The Staphylococcus aureus AirSR two-component system mediates reactive oxygen species resistance via transcriptional regulation of staphyloxanthin production. Infect Immun 85:e00838-16. doi:10.1128/IAI.00838-16 27872240 PMC5278164

[32] Shukla P, Khodade VS, SharathChandra M, Chauhan P, Mishra S, Siddaramappa S, Pradeep BE, Singh A, Chakrapani H. 2017. “On demand” redox buffering by H_2_S contributes to antibiotic resistance revealed by a bacteria-specific H_2_S donor. Chem Sci 8:4967–4972. doi:10.1039/c7sc00873b 28959420 PMC5607856

[33] Chen C, Han P, Yan W, Wang S, Shi X, Zhou X, Desneux N, Gao X. 2018. Uptake of quercetin reduces larval sensitivity to lambda-cyhalothrin in Helicoverpa armigera. J Pest Sci 91:919–926. doi:10.1007/s10340-017-0933-1

[34] Ruiz C, Levy SB. 2014. Regulation of acrAB expression by cellular metabolites in Escherichia coli. J Antimicrob Chemother 69:390–399. doi:10.1093/jac/dkt352 24043404 PMC3886929

[35] Lee HH, Molla MN, Cantor CR, Collins JJ. 2010. Bacterial charity work leads to population-wide resistance. Nature 467:82–85. doi:10.1038/nature09354 20811456 PMC2936489

[36] Kuroyanagi T, Bulasag AS, Fukushima K, Ashida A, Suzuki T, Tanaka A, Camagna M, Sato I, Chiba S, Ojika M, Takemoto D. 2022. Botrytis cinerea identifies host plants via the recognition of antifungal capsidiol to induce expression of a specific detoxification gene. PNAS Nexus 1:pgac274. doi:10.1093/pnasnexus/pgac274 36712336 PMC9802192

[37] Dietrich LEP, Price-Whelan A, Petersen A, Whiteley M, Newman DK. 2006. The phenazine pyocyanin is a terminal signalling factor in the quorum sensing network of Pseudomonas aeruginosa. Mol Microbiol 61:1308–1321. doi:10.1111/j.1365-2958.2006.05306.x 16879411

[38] Meirelles LA, Newman DK. 2018. Both toxic and beneficial effects of pyocyanin contribute to the lifecycle of Pseudomonas aeruginosa. Mol Microbiol 110:995–1010. doi:10.1111/mmi.14132 30230061 PMC6281804

[39] Meirelles LA, Perry EK, Bergkessel M, Newman DK. 2021. Bacterial defenses against a natural antibiotic promote collateral resilience to clinical antibiotics. PLoS Biol 19:e3001093. doi:10.1371/journal.pbio.3001093 33690640 PMC7946323

[40] Andrade AC, Van Nistelrooy JG, Peery RB, Skatrud PL, De Waard MA. 2000. The role of ABC transporters from Aspergillus nidulans in protection against cytotoxic agents and in antibiotic production. Mol Gen Genet 263:966–977. doi:10.1007/pl00008697 10954082

[41] Morschhäuser J. 2010. Regulation of multidrug resistance in pathogenic fungi. Fungal Genet Biol 47:94–106. doi:10.1016/j.fgb.2009.08.002 19665571

[42] Hayashi K, Schoonbeek H, Sugiura H, De Waard MA. 2001. Multidrug resistance in Botrytis cinerea associated with decreased accumulation of the azole fungicide oxpoconazole and increased transcription of the ABC transporter gene BcatrD. Pestic Biochem Physiol 70:168–179. doi:10.1006/pest.2001.2548

[43] Tscherner M, Schwarzmüller T, Kuchler K. 2011. Pathogenesis and antifungal drug resistance of the human fungal pathogen Candida glabrata. Pharmaceuticals 4:169–186. doi:10.3390/ph4010169

[44] Oliver A, Cantón R, Campo P, Baquero F, Blázquez J. 2000. High frequency of hypermutable Pseudomonas aeruginosa in cystic fibrosis lung infection. Science 288:1251–1254. doi:10.1126/science.288.5469.1251 10818002

[45] Lewis K, Shan Y. 2017. Why tolerance invites resistance. Science 355:796. doi:10.1126/science.aam7926 28232537

[46] Cools HJ, Hawkins NJ, Fraaije BA. 2013. Constraints on the evolution of azole resistance in plant pathogenic fungi. Plant Pathology 62:36–42. doi:10.1111/ppa.12128

[47] Ma C, Niu R, Huang T, Shao L-W, Peng Y, Ding W, Wang Y, Jia G, He C, Li C-Y, He A, Liu Y. 2019. N6-methyldeoxyadenine is a transgenerational epigenetic signal for mitochondrial stress adaptation. Nat Cell Biol 21:319–327. doi:10.1038/s41556-018-0238-5 30510156

[48] Walsh TK, Heckel DG, Wu Y, Downes S, Gordon KHJ, Oakeshott JG. 2022. Determinants of insecticide resistance evolution: comparative analysis among heliothines. Annu Rev Entomol 67:387–406. doi:10.1146/annurev-ento-080421-071655 34995087

[49] Guo Z, Guo L, Bai Y, Kang S, Sun D, Qin J, Ye F, Wang S, Wu Q, Xie W, Yang X, Crickmore N, Zhou X, Zhang Y. 2023. Retrotransposon-mediated evolutionary rewiring of a pathogen response orchestrates a resistance phenotype in an insect host. Proc Natl Acad Sci USA 120:e2300439120. doi:10.1073/pnas.2300439120 36996102 PMC10083559

[50] Thiel G, Rössler OG. 2017. Resveratrol regulates gene transcription via activation of stimulus-responsive transcription factors. Pharmacol Res 117:166–176. doi:10.1016/j.phrs.2016.12.029 28012964

[51] Liu Z, Rossi JM, Myers LC. 2018. Candida albicans Zn cluster transcription factors Tac1 and Znc1 are activated by farnesol to upregulate a transcriptional program including the multidrug efflux pump CDR1. Antimicrob Agents Chemother 62:e00968-18. doi:10.1128/AAC.00968-18 30104273 PMC6201089

[52] Schillig R, Morschhäuser J. 2013. Analysis of a fungus‐specific transcription factor family, the Candida albicans zinc cluster proteins, by artificial activation. Mol Microbiol 89:1003–1017. doi:10.1111/mmi.12327 23844834

[53] Hirakawa H, Inazumi Y, Masaki T, Hirata T, Yamaguchi A. 2005. Indole induces the expression of multidrug exporter genes in Escherichia coli. Mol Microbiol 55:1113–1126. doi:10.1111/j.1365-2958.2004.04449.x 15686558

[54] Song Y, Zhang Z, Chen L, He L, Lu H, Ren Y, Mu W, Liu F, Pests I. 2016. Baseline sensitivity of Botrytis cinerea to the succinate dehydrogenase inhibitor isopyrazam and efficacy of this fungicide. Plant Dis 100:1314–1320. doi:10.1094/PDIS-10-15-1220-RE 30686199

[55] Adnan M, Hamada MS, Hahn M, Li GQ, Luo CX. 2019. Fungicide resistance of Botrytis cinerea from strawberry to procymidone and zoxamide in Hubei, China. Phytopathol Res 1:1–12. doi:10.1186/s42483-019-0024-8

[56] Cheng X, Man X, Wang Z, Liang L, Zhang F, Wang Z, Liu P, Lei B, Hao J, Liu X. 2020. Fungicide SYP-14288 inducing multidrug resistance in Rhizoctonia solani. Plant Dis 104:2563–2570. doi:10.1094/PDIS-01-20-0048-RE 32762501

[57] Schmittgen TD, Livak KJ. 2008. Analyzing real-time PCR data by the comparative CT method. Nat Protoc 3:1101–1108. doi:10.1038/nprot.2008.73 18546601

[58] Bian Y, Guo G, Liu F, Chen X, Wang Z, Hou T. 2020. Meptyldinocap and azoxystrobin residue behaviors in different ecosystems under open field conditions and distribution on processed cucumber. J Sci Food Agric 100:648–655. doi:10.1002/jsfa.10059 31577839

